# Successful Treatment of Refractory Ulcerative Colitis With 5-Aminosalicylic Acid Intolerance and Biologic Therapy Resistance Using Combined Granulocyte and Monocyte Adsorptive Apheresis

**DOI:** 10.7759/cureus.77641

**Published:** 2025-01-18

**Authors:** Tomotaka Tanaka, Daiki Hirano, Syohei Ishimaru, Keiko Arataki

**Affiliations:** 1 Department of Gastroenterology, Tsuchiya General Hospital, Hiroshima, JPN

**Keywords:** biologic therapy resistance, golimumab, granulomonocytapheresis, intolerance to 5-aminosalicylates, ulcerative colitis

## Abstract

We report the case of a 37-year-old male patient diagnosed with moderate left-sided ulcerative colitis (UC). Initial therapy with 5-aminosalicylic acid (5-ASA) was terminated within days due to exacerbation of symptoms, leading to a diagnosis of 5-ASA intolerance. Although induction of remission was achieved with prednisolone, the patient developed steroid dependency. Treatment with vedolizumab and ustekinumab subsequently failed to achieve clinical or endoscopic improvement. Intensive granulocyte and monocyte apheresis (GMA) was introduced, successfully inducing remission. However, during maintenance therapy with GMA, the patient experienced a relapse. Initiation of golimumab yielded suboptimal results, necessitating a combination therapy involving prednisolone and reintensified intensive GMA. This multimodal approach successfully achieved remission induction and maintenance. This case highlights the potential utility of intensive GMA in combination with golimumab and prednisolone for the management of refractory UC, particularly in patients with 5-ASA intolerance and failure of multiple biologic agents. A brief review of the relevant literature is included.

## Introduction

5-Aminosalicylic acid (5-ASA) is the first-line therapy for ulcerative colitis (UC). However, about 10% of patients are reported to have intolerance to 5-ASA preparations [1]. Lack of response to baseline 5-ASA treatment presents a significant clinical challenge in induction and maintenance therapy for UC. Now, no established treatment guidelines exist for UC patients intolerant to 5-ASA. This case involved a patient who exhibited intolerance to 5-ASA and secondary loss of response (LOR) to vedolizumab (VED), as well as primary non-response to ustekinumab (UST), resulting in refractory UC that was difficult to manage. VED has been reported to show diminished efficacy in approximately 40% of cases [2], and in the case of UST, it has been reported that its response is suboptimal when used as a second-line therapy [3]. We report a case of refractory UC intolerant to 5-ASA and unresponsive to multiple biologics, where induction and maintenance of remission were achieved with a combination of granulocyte and monocyte adsorptive apheresis (GMA), golimumab (GLM), and prednisolone (PSL). A brief review of the literature is also provided.

## Case presentation

A 37-year-old man presented in April 2022 with complaints of hematochezia lasting for six months, more than 10 bowel movements per day, and abdominal discomfort. Medical and family history showed no significant findings.

**Figure 1:**
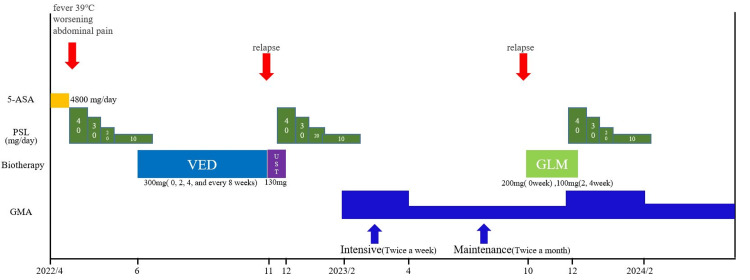
Clinical course VED: Vedolizumab; GLM: golimumab; GMA: granulocyte and monocyte adsorptive apheresis; PSL: prednisolone

**Figure 2:**
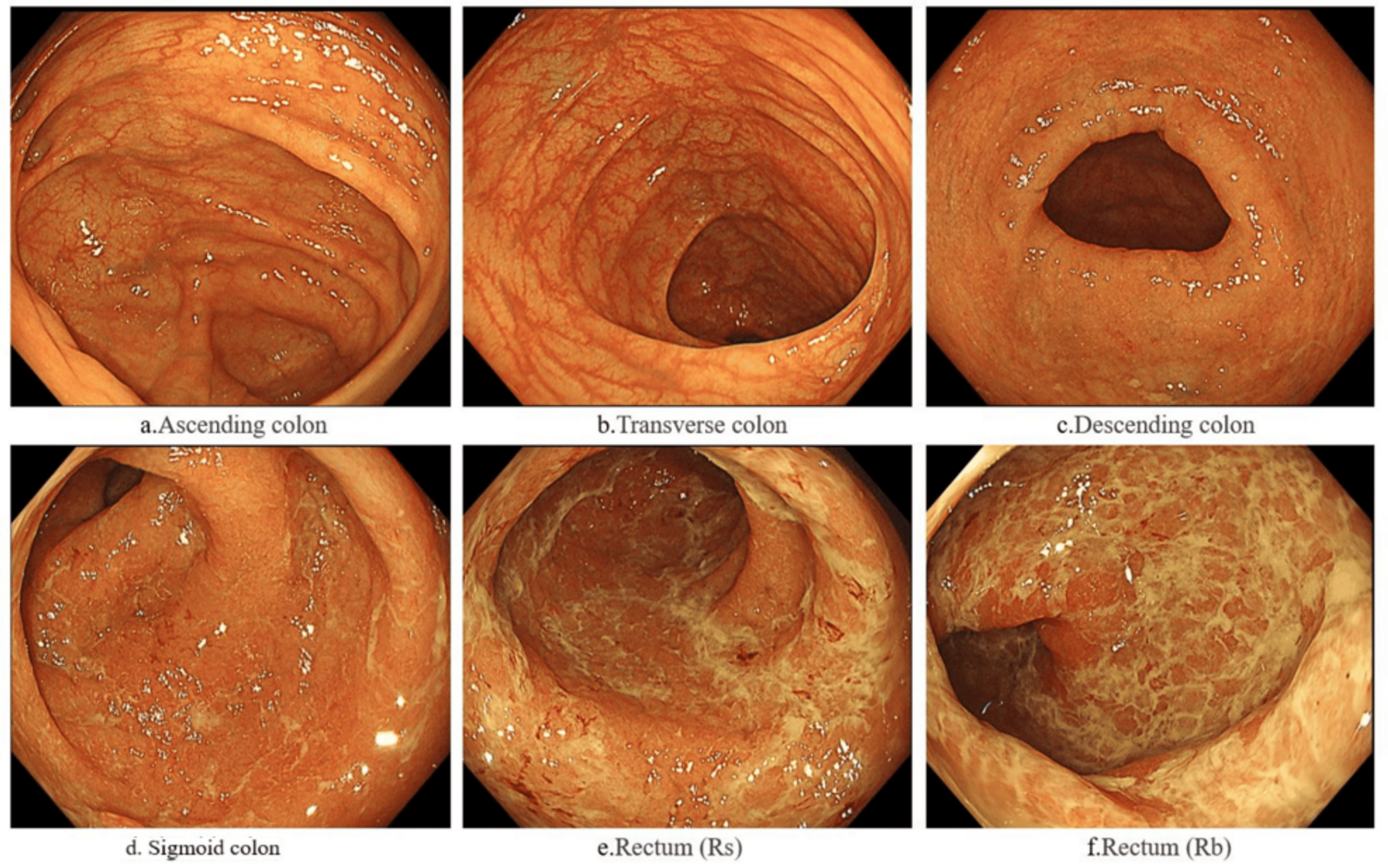
Colonoscopy photos a: Ascending colon; b: Transverse colon; c: Loss of the vascular pattern and mild erythema were observed in the descending colon; d-f: Numerous white exudates extending from the rectum to the descending colon were observed.

The clinical course is shown in Figure 1. Blood tests (Table 1) revealed mild leukocytosis and elevated C-reactive protein (CRP) levels, with no other abnormalities. Stool culture tests (Table 1) were negative. Colonoscopy (Figure 2) showed loss of vascular pattern, mild erythema, and numerous white exudates extending from the rectum to the descending colon, leading to a diagnosis of left-sided colitis-type UC. The biopsy of the sigmoid colon and rectum showed no dysplasia in the intestinal epithelium. Regenerative changes were present with moderate chronic inflammatory cell infiltration. The glands were slightly irregular and atrophic, but no crypt abscesses were observed. The patient's disease severity was classified as moderate, with a leucine-rich alpha-2 glycoprotein (LRG) level of 18.8 µg/mL and a Mayo score of 9.

**Table 1:** Test results NUDT-15: Nudix hydrolase 15

Parameters	Results	Reference values
WBC	10300/μl	3300-8600/μl
Neu	51.70%	45.2-68.8%
Lym	37.70%	26.8-43.8%
Mon	8.90%	2.7-7.9%
Eo	1.20%	0.0-10.0%
Ba	0.50%	0.0-5.0%
RBC	481x104/μl	435-555/μl
Hgb	15.6g/dl	13.7-16.8/dl
Hct	46.50%	40.7-50.1%
Plt	38.6x104/μl	15.8-34.8/μl
T.P.	7.6g/dl	6.6-8.1/dl
Alb	4.2g/dl	4.1-5.1/dl
AST	24U/l	13-30U/l
ALT	28U/l	10-42U/l
ChE	332U/l	240-486U/l
LDH	210U/l	124-222U/l
T-Bil	0.6mg/dl	0.4-1.5mg/dl
BUN	18mg/dl	8-20mg/dl
Cre	0.67mg/dl	0.65-1.07mg/dl
Na	138.6mEq/l	138-145mEq/l
K	4.6mEq/l	3.6-4.8mEq/l
Cl	102mEq/l	101-108mEq/l
CRP	3.02mg/dl	0.14≧
LRG (leucine-rich alpha2 glycoprotein)	18.8 μg/ml	16.0＞
Stool culture	negative	-
Mayo score	9 point	-
NUDT-15	Arg/Cys	-

Treatment was initiated with 5-ASA at 4800 mg/day. However, 3-4 days after starting 5-ASA, the patient developed fever (39°C) and severe abdominal pain. Suspecting 5-ASA intolerance, the drug was discontinued, and PSL 40 mg/day was started. Symptoms improved rapidly with PSL, which was subsequently tapered. However, symptoms recurred when PSL was reduced to 10 mg/day, suggesting steroid dependence. NUDT-15 was Arg/Cys, the patient decided, after consultation, not to proceed with azathioprine treatment. Similarly, the patient was concerned about potential side effects, such as infusion reactions, and opted not to proceed with infliximab treatment.

VED at 300 mg was initiated as induction therapy, administered at zero, two, four, and every eight weeks. Initially, symptoms improved; however, from the sixth dose of VED, bowel frequency and hematochezia increased, indicating secondary LOR to VED. UST at 130 mg was then administered but showed no effect, leading to its discontinuation. PSL 40 mg was reintroduced, which reduced bowel frequency, abdominal pain, and hematochezia.

**Figure 3:**
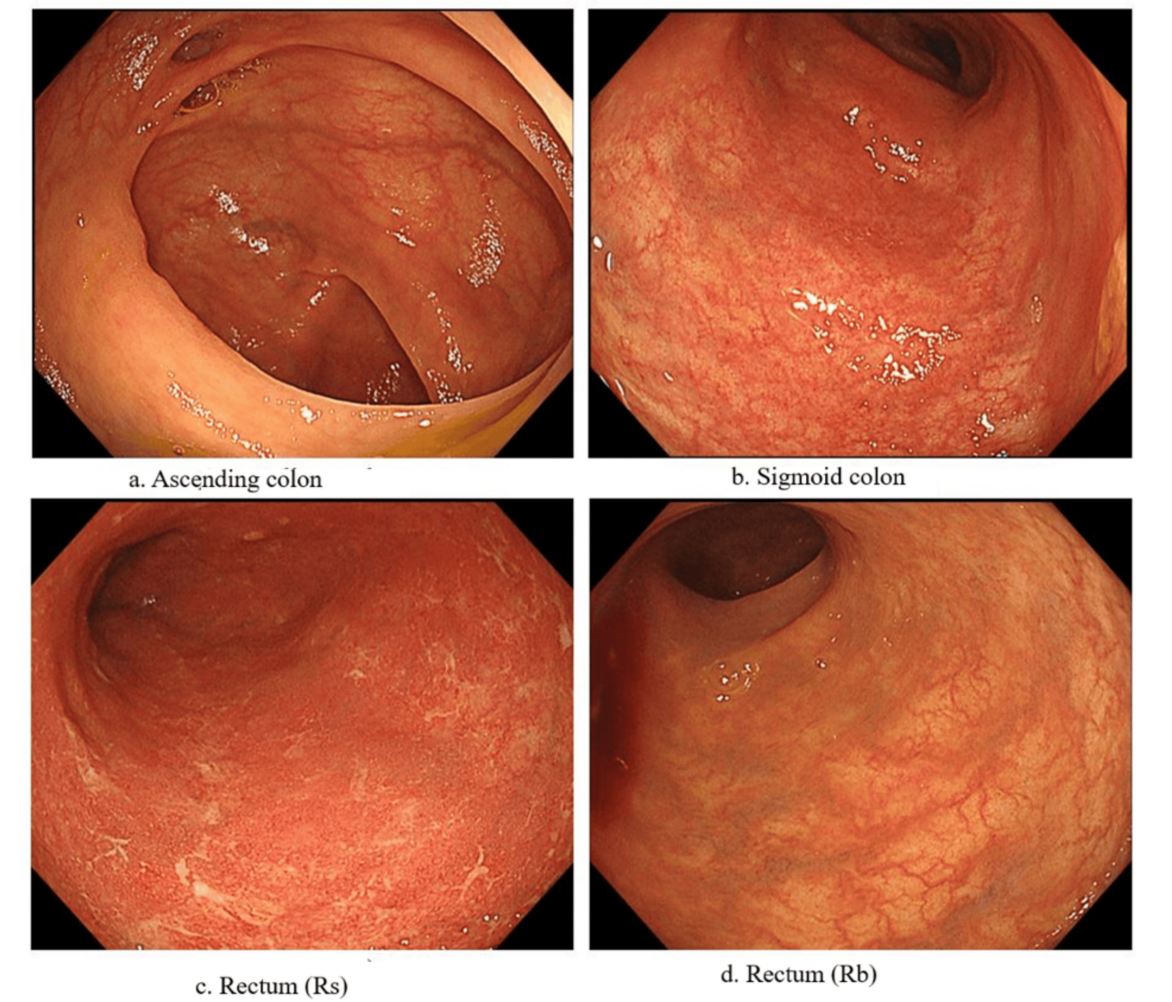
Colonoscopy photos The vascular pattern has become visible, and mucus has decreased in the rectal mucosa and sigmoid colon compared to that before treatment.

The patient underwent intensive GMA (twice weekly for a total of 10 sessions) while maintaining PSL 10 mg. Symptoms improved, and PSL was discontinued, transitioning to maintenance GMA therapy (twice monthly). After six months of maintenance GMA, symptoms recurred, including abdominal pain, hematochezia, and increased bowel movements. Despite continuing GMA, GLM was initiated at zero week (200 mg), two weeks (100 mg), and four weeks (100 mg) without significant improvement. Intensive GMA combined with PSL 40 mg was reinstated, followed by gradual tapering of PSL and continuation of maintenance GMA (twice monthly). The patient achieved sustained remission for one year (Figure 3).

## Discussion

5-ASA formulations are widely recognized as standard therapeutic agents for both remission induction and maintenance in patients with active UC. However, 5-ASA itself may induce symptoms resembling disease exacerbation, including abdominal pain, fever, diarrhea, and hematochezia, a phenomenon referred to as 5-ASA intolerance [1]. The reported incidence of 5-ASA intolerance varies among studies. Fukushima et al. and Motoya et al. reported an incidence of approximately 2% [4,5], whereas Hiraoka et al. observed that approximately 11% of UC patients exhibited intolerance, with a year-on-year increase from 5.3% in 2007-2010 to 9.1% in 2011-2013 and 16.2% in 2014-2016 [1].

5-ASA intolerance can be classified into two categories: a narrow definition, characterized by adverse effects due to reduced metabolic capacity without immune involvement, and a broader definition, which encompasses immune-mediated hypersensitivity reactions. According to Fukushima et al., the mean time to onset of intolerance was 7.7 days, with a dose-dependent relationship [4]. Although the precise mechanisms remain unclear, management strategies typically involve drug discontinuation or substitution. Motoya et al. reported that the drug lymphocyte stimulation test (DLST) demonstrated a 47.6% positivity rate in cases of intolerance, indicating high specificity despite low sensitivity [5]. Clinicians should suspect 5-ASA intolerance when clinical trajectories deviate unexpectedly after initiation, and DLST may serve as a valuable diagnostic tool.

Differentiating 5-ASA intolerance from genuine disease exacerbation with mucosal involvement in UC patients can be challenging. Delayed diagnosis may result in severe consequences, including colectomy [6]. Early intervention with corticosteroids or biologic agents should be considered in such cases.

In the present case, corticosteroids were employed for remission induction and maintenance due to 5-ASA intolerance. However, steroid tapering led to relapse, suggesting steroid dependency. For steroid-dependent or refractory UC cases, biologic agents are typically recommended. Although VDZ demonstrated partial efficacy in this case, UST was ineffective and discontinued. Notably, GLM, in combination with PSL and GMA, appeared to contribute to remission induction in this refractory UC case.

The efficacy of GMA in cases of secondary LOR to biologics has been reported in the literature. Several mechanisms may underlie the beneficial effects of combination therapy. One proposed mechanism involves GMA-mediated improvements in drug trough levels, reductions in anti-drug antibodies, or both [7-9]. Notably, GMA has been reported to elevate serum trough levels of infliximab in cases of secondary LOR [10]. Shimoyama et al. demonstrated that GMA suppresses cytokine production by evaluating serum inflammatory cytokine levels before and after treatment [11]. Tanida et al. reported that a combination of tofacitinib, a JAK inhibitor, and intensive GMA achieved a clinical remission rate of 71.4% at 10 weeks in seven patients [12].

Saniabadi et al. hypothesized that the combination of GMA and tofacitinib synergistically downregulates systemic inflammatory cytokines and adhesion molecule expression on activated granulocytes (via GMA) and locally suppresses inflammatory cytokines in the intestinal microenvironment (via tofacitinib), thereby facilitating rapid and robust clinical remission [13]. GMA has also been reported to reduce activated myeloid-derived leukocytes, which are primary sources of inflammatory cytokines, indirectly mitigating intestinal mucosal damage [13].

Nakamura et al. demonstrated the efficacy of combining VDZ with GMA in refractory UC cases [14]. Similarly, Tanida et al. reported that intensive GMA combined with adalimumab achieved a remission rate of 47% at week 10 and 45% at week 52 in steroid-dependent or resistant moderate-to-severe UC patients [15]. These findings collectively suggest that adjunctive GMA therapy may enhance the efficacy of biologics for remission induction in refractory UC cases.

In cases such as the present one, where multiple biologics fail, the combination of GMA with other therapeutic modalities may serve as a valuable strategy, highlighting the potential for repositioning GMA in the treatment paradigm for refractory UC. Further accumulation of case data and clinical studies will be essential to validate these findings.

## Conclusions

GMA is approved for both remission induction and maintenance therapy in refractory UC cases. For patients with special circumstances, such as 5-ASA intolerance or resistance to multiple biologic agents, GMA, either as monotherapy or in combination with other agents, represents a promising and safe therapeutic option for achieving and maintaining remission. Moreover, there have been no previous case reports of successful combination therapy of golimumab and GMA for refractory UC, suggesting the possibility of future combination therapy with GMA.

## References

[1] Hiraoka S, Fujiwara A, Toyokawa T (2021). Multicenter survey on mesalamine intolerance in patients with ulcerative colitis. J Gastroenterol Hepatol.

[2] Peyrin-Biroulet L, Danese S, Argollo M (2019). Loss of response to vedolizumab and ability of dose intensification to restore response in patients with Crohn's disease or ulcerative colitis: a systematic review and meta-analysis. Clin Gastroenterol Hepatol.

[3] Singh S, George J, Boland BS, Vande Casteele N, Sandborn WJ (2018). Primary non-response to tumor necrosis factor antagonists is associated with inferior response to second-line biologics in patients with inflammatory bowel diseases: a systematic review and meta-analysis. J Crohns Colitis.

[4] Fukushima T, Nakajima K, Henmi H (2014). Desensitization therapy for mesalazine-intolerant patients with inflammatory bowel disease (Article in Japanese). J Jpn Colonproctol.

[5] Motoya S, Shimodate Y, Tanaka H Mesalamine tolerance of ulcerative colitis. IBD Res.

[6] Ding H, Liu XC, Mei Q, Xu JM, Hu XY, Hu J (2014). Ulcerative colitis flair induced by mesalamine suppositories hypersensitivity. World J Gastroenterol.

[7] Rodríguez-Lago I, Sempere L, Gutiérrez A (2019). Granulocyte-monocyte apheresis: an alternative combination therapy after loss of response to anti-TNF agents in ulcerative colitis. Scand J Gastroenterol.

[8] Rodríguez-Lago I, Benítez JM, Sempere L, Sáez-González E, Barreiro-de Acosta M, de Zárate JO, Cabriada JL (2019). The combination of granulocyte-monocyte apheresis and vedolizumab: a new treatment option for ulcerative colitis?. J Clin Apher.

[9] Sáez-González E, Aguas M, Huguet JM, Nos P, Beltrán B (2018). Combination therapy with cytapheresis plus vedolizumab in a corticosteroid-dependent patient with ulcerative colitis and previous ANTI-TNF-α drug failure. Dig Liver Dis.

[10] Yokoyama Y, Kamikozuru K, Watanabe K, Nakamura S (2018). Inflammatory bowel disease patients experiencing a loss of response to infliximab regain long-term response after undergoing granulocyte/monocyte apheresis: a case series. Cytokine.

[11] Shimoyama T, Sawada K, Hiwatashi N (2001). Safety and efficacy of granulocyte and monocyte adsorption apheresis in patients with active ulcerative colitis: a multicenter study. J Clin Apher.

[12] Tanida S, Ozeki K, Mizoshita T (2020). Combination therapy with tofacitinib plus intensive granulocyte and monocyte adsorptive apheresis as induction therapy for refractory ulcerative colitis. J Clin Med Res.

[13] Saniabadi AR, Tanaka T, Ohmori T, Sawada K, Yamamoto T, Hanai H (2014). Treating inflammatory bowel disease by adsorptive leucocytapheresis: a desire to treat without drugs. World J Gastroenterol.

[14] Nakamura M, Yamamura T, Maeda K (2020). Refractory ulcerative colitis improved by scheduled combination therapy of vedolizmab and granulocyte and monocyte adsorptive apheresis. Intern Med.

[15] Tanida S, Mizoshita T, Nishie H (2015). Combination therapy with adalimumab plus intensive granulocyte and monocyte adsorptive apheresis in patients with refractory ulcerative colitis. J Clin Med Res.

